# Iatrogenic Lower Extremity Subcutaneous Emphysema after Prolonged Robotic-Assisted Hysterectomy

**DOI:** 10.1155/2015/860719

**Published:** 2015-12-16

**Authors:** Monica Hagan Vetter, Chelsea Mutscheller, Joel Cardenas-Goicoechea

**Affiliations:** ^1^Department of Obstetrics/Gynecology, The Ohio State University, 5th Floor, 395 West 12th Avenue, Columbus, OH 43210, USA; ^2^The Mark H. Zangmeister Center, 3100 Plaza Properties Boulevard, Columbus, OH 43219, USA

## Abstract

Subcutaneous emphysema is a known complication of carbon dioxide insufflation, an essential component of laparoscopy. The literature contains reports of hypercarbia, pneumothorax, or pneumomediastinum. However, isolated lower extremity subcutaneous emphysema remains a seldom-reported complication. We report a case of unilateral lower extremity subcutaneous emphysema following robotic-assisted hysterectomy, bilateral salpingooophorectomy, staging, and anterior/posterior colporrhaphy for carcinosarcoma and vaginal prolapse. On postoperative day 1, the patient developed tender crepitus and bruising of her right ankle. Radiography confirmed presence of subcutaneous air. Vital signs and laboratory findings were unremarkable. Her symptoms spontaneously improved over time, and she was discharged in good condition on day 2. In stable patients with postoperative extremity swelling or pain with crepitus on exam, the diagnosis of iatrogenic subcutaneous emphysema must be considered.

## 1. Introduction

Robotic-assisted surgery is becoming more common in the fields of general surgery, urology, and gynecology. At least 1.5 million procedures have been performed robotically over the last 10 years [1]. In gynecology, robotic-assisted surgery is used for both benign and malignant indications. Essential components of robotic surgery include carbon dioxide insufflation and use of steep Trendelenburg. Although insufflation with carbon dioxide is generally considered safe, there are small but important risks of developing complications such as subcutaneous emphysema, hypercarbia, pneumothorax, pneumomediastinum, and carbon dioxide embolism [2–5]. In one study of 968 laparoscopic cases, 5.5% of cases were complicated by hypercarbia, 2.3% by subcutaneous emphysema, and 1.9% for pneumothorax/pneumomediastinum [6]. We report a rare case of unilateral lower extremity iatrogenic subcutaneous emphysema following robotic comprehensive staging of uterine carcinosarcoma and anterior/posterior colporrhaphy.

## 2. Clinical Case

An 89-year-old G3P3003 presented to the outpatient office with complaints of postmenopausal bleeding and pelvic organ prolapse for the past 12 months. Physical exam confirmed anterior and posterior vaginal prolapse while endometrial biopsy revealed uterine carcinosarcoma. Her past medical history included hypertension, hypothyroidism, COPD, type 2 diabetes, hyperlipidemia, and obesity (BMI of 31 kg/m^2^). Prior abdominal surgery was significant for an appendectomy via laparotomy in childhood. As the patient had a good functional status (ECOG status of 1) as well as symptomatic prolapse and bleeding, the decision was made to proceed with surgery. She underwent robotic hysterectomy, bilateral salpingooophorectomy, pelvic lymph node dissection, and omentectomy with anterior/posterior colporrhaphy for carcinosarcoma of the uterus and pelvic organ prolapse. The total procedure including the robotic portion and colporrhaphy lasted 260 minutes and was uncomplicated. Time of sustained pneumoperitoneum was 150 minutes with an intra-abdominal pressure of 14 mm Hg. As the patient tolerated both the steep Trendelenburg positioning and the sustained pneumoperitoneum, the intra-abdominal pressure of 14 mm Hg was maintained throughout the case for optimal visualization of the enlarged uterus. Five laparoscopic ports were used.

**Figure 1:**
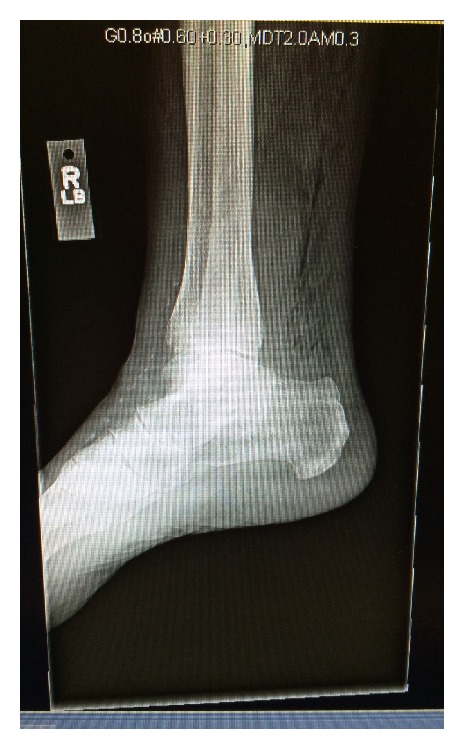
Radiograph of right ankle. There is diffuse subcutaneous air noted posteriorly to the tibia.

On postoperative day 1, the patient reported new onset of pain in her right lower extremity. The patient denied any prior trauma to this area. A physical exam revealed that right lower extremity bruising and crepitus were noted at the lateral malleolus extending to the dorsal surface and tracing cephalad to the knee. A radiograph of the right ankle was obtained which showed extensive soft tissue gas (Figure 1). Final radiologic impression noted diffuse subcutaneous air posterior to the tibia concerning for possible necrotizing fasciitis. During this time, she was noted to be afebrile with a maximum temperature of 99.4°F. Her pulse remained in the range of 80–90 BPM, and she was mildly hypertensive at 148/92. Labs obtained revealed a WBC of 8.1 × 10^9^/L, lactic acid of 1.4 mg/dL, total creatinine kinase of 214 U/L, and creatinine of 0.72 mg/dL. A lower extremity Doppler was negative for deep venous thrombus. Infectious disease and general surgery were consulted with both services agreeing that there was no clinical evidence of necrotizing fasciitis.

Upon further investigation, the patient was found to have a hernia at the location of her prior appendectomy incision. It was ultimately determined that the existing disruption of the fascia due to this incisional hernia had allowed a communication between the peritoneum and the right lower extremity causing carbon dioxide to escape the abdominal compartment. Compression stockings were then placed on the patient's leg. Cold compresses were also intermittently used on the extremity to decrease both inflammation and pain.

Postoperatively, the patient did well and enjoyed an otherwise uneventful recovery. She was discharged to home in good condition on postoperative day 3. She was seen in the office three weeks after discharge where she was doing well. There were no crepitus or bruising at that time. She also reported that her prolapse symptoms were resolved. Upon discussion with the patient and her family, the patient elected for no adjuvant therapy for the treatment of her carcinosarcoma. At the time of her six-month follow-up the patient was alive and without evidence of disease.

## 3. Discussion

Subcutaneous emphysema is the consequence of air introduction or other gases into soft tissue. Studies of laparoscopic surgery approaches in which carbon dioxide insufflation and steep Trendelenburg were utilized have demonstrated subcutaneous emphysema in upper extremities, pneumothorax, and pneumomediastinum. Predisposing factors include operative time longer than 200 minutes, use of more than 5 laparoscopic ports, and older age [6]. Our patient's risk factors for the development of her lower extremity subcutaneous emphysema included operative time, age, and number of ports. This patient also had additional risk from the presence of the fascial defect from her prior appendectomy. While this case report features iatrogenic subcutaneous emphysema, subcutaneous emphysema and associated crepitus may have other causes such as trauma or necrotizing infections.

Necrotizing infections are typically associated with gas-forming bacteria such as* Clostridium* and typically involve the fascia and subcutaneous tissues [7]. Crepitus and sensory changes are the first symptoms of these infections, which warrant immediate surgical intervention. Laboratory findings include acidosis, leukocytosis, increased creatinine kinase, or creatinine due to extensive tissue breakdown. In the presented patient, all laboratory findings were within normal limits suggesting that her subcutaneous emphysema had a more benign cause. The development of this finding acutely following her operation was suspicious for a temporal relationship between the surgical pneumoperitoneum and development of lower extremity subcutaneous emphysema. The defect in the abdominal fascia secondary to the patient's existing incisional hernia was the most likely route of gas entry into the leg.

To our knowledge, this is the second reported case of isolated unilateral lower extremity subcutaneous emphysema following robotic surgery [8]. In the previously reported case, a 71-year-old female presented on postoperative day 2 following a robotic supracervical hysterectomy, robotic sacral colpopexy, and cystoscopy for uterovaginal prolapse with complaints of bilateral leg pain and swelling. On clinical exam she had significant tenderness and crepitus. Lower extremity Doppler was performed to evaluate deep venous thromboembolism and showed significant subcutaneous air. It was felt that gas entered the lower extremities through the femoral canal along the vascular sheaths as this patient did not have any recognized fascial defects. The patient was discharged home with leg compression and had symptom resolution by postoperative day 13.

Techniques for prevention of this complication could include minimization of time in Trendelenburg and use of the lowest number of ports possible. Additionally, a study by Lee et al. showed that the incidence of subcutaneous emphysema increased with maintenance of higher intraabdominal pressures during laparoscopy [9]. Therefore, utilization of the lowest intra-abdominal pressure during surgery could minimize the risk of this complication. In patients with a history of prior abdominal surgery, inspection of prior surgical sites could reveal incisional hernias identifying possible routes of carbon dioxide escape. Due to the rarity of this complication, it is unknown if there is a difference in risks with procedures performed robotically or with traditional laparoscopy. As there are typically a fewer number of ports used in traditional laparoscopy in comparison with robotic surgery, this may decrease the risk of the development of iatrogenic extremity subcutaneous emphysema. This must, however, be weighed against potentially increased operating times with traditional laparoscopy versus robotic surgery.

This could alert surgeons to patients at potentially higher risk of iatrogenic extremity subcutaneous emphysema; however it is unclear at this time whether surgical correction of the defect may have a clinical impact. As it appears that there is a low morbidity associated with this isolated subcutaneous emphysema if it should occur, the risks of repairing these incisional hernias should be carefully considered.

As minimal invasive surgery for both benign and malignant indications increases in conjunction with trend towards shorter hospital admission, complications associated with carbon dioxide insufflation should be kept in mind when caring for patients immediately after operation. It is important that the patient be evaluated for potential life-threatening infections. However, in patients with complaints of extremity swelling or pain with crepitus on exam with stable vital signs and laboratory findings, the diagnosis of benign isolated subcutaneous emphysema must be on the differential.
